# “We find that…” changing patterns of epistemic positioning in research writing

**DOI:** 10.3389/fpsyg.2025.1634848

**Published:** 2025-09-26

**Authors:** Yanfang Yang, Xuan Guo

**Affiliations:** School of Foreign Languages, Xinxiang Institute of Engineering, Xinxiang, China

**Keywords:** epistemic positioning, hedges and boosters, diachronic change, disciplinary variation, research writing history

## Abstract

**Introduction:**

Epistemic positioning refers to the writer’s commitment to the truth of a proposition and assessment of its potential impact on readers. Despite its importance, little attention has been paid to how writers make epistemic judgments across disciplines over time.

**Methods:**

Drawing on Hyland and Zou’s taxonomies of hedges and boosters, we analyzed 240 research articles from education, history, mechanical engineering, and physics, covering three periods (1960, 1990, and 2020).

**Results:**

Our findings show that epistemic positioning has significantly decreased across all four disciplines over time, with writers increasingly preferring less use of epistemic markers in pursuit of an objective, data-based, and scientific style.

**Discussion:**

These results suggest a disciplinary shift in research writing practices and have important implications for raising students’ and novice academic writers’ awareness of evolving knowledge discourses shaped by changing societies.

## 1 Introduction

Successful academic writing partly lies in the writer’s ability to balance conviction by investing their statements with the confidence or uncertainty of knowledge, to make their work convincing, and to gain the acceptance of their colleagues and readers (Hyland, 2000). We referred to the expressions of doubt or certainty of knowledge as epistemic positioning based on Hyland and Guinda (2012), which is marked by hedges and boosters. These devices are important communicative strategies for the writer to strengthen or weaken the force of their statement. They help the writer to convey both his epistemic and affective meanings; that is to say, they carry not only the writer’s degree of confidence in the truth of the information he provides but also an attitude to the readers (Hyland, 2004). Writers need to consider that their claims are at risk of being negated by the readers. Therefore, writers must carefully craft their statements to achieve effective persuasion.

An increasing number of studies have explored how epistemic positioning is conveyed, focusing on variations across languages and cultures (Hu and Cao, 2011; Mur-Dueñas, 2021), between student and professional writing (Aull et al., 2017; Qiu et al., 2024), across genres and disciplines (Bondi, 2005), among languages and disciplines (Deng and He, 2023; Hu and Cao, 2015), and across disciplines over time (Deng et al., 2021; Hyland and Jiang, 2016, 2018). However, little attention has been paid to the extent to which writers make epistemic judgments that vary across disciplines over time. In this study, we aimed to explore this issue using Hyland and Zou (2021) taxonomies of hedges and boosters. Based on a corpus of 1.3 million words taken from 240 research articles from four disciplines at three distinct periods, we seek to address the following questions:

(1) What are the forms and frequency of epistemic positioning in research writing?
(2) What are the functions of epistemic positioning in shaping academic persuasion?
(3) To what extent do the forms and functions of epistemic positioning vary across disciplines and time?

## 2 Literature review

### 2.1 Definition of epistemic positioning

Epistemic positioning, also called evidentiality (Chafe and Nichols, 1986) or epistemic stance (Biber et al., 1999), refers to the writer’s commitment to the reliability of the propositions *he* or *she* provides and the assessment of their potential impact on the readers (Hyland, 2005b). It is commonly expressed through hedges and boosters, which are the focus of interactional metadiscourse in academic writing.

Hedges are linguistic features that make things fuzzy (Lakoff, 1973), realized through words such as *might, perhaps, maybe, seem, and indicate,* and phrases such as *in my view, on the whole, in most cases, and to some extent*. The use of hedges signals the writer’s unwillingness to make an explicit and full commitment to the truth of the propositions *he* or *she* presented (Hyland, 1998b). They are crucial in the rhetorical construction of knowledge, as they allow writers to open a discursive space, express their opinions with caution, and mark their claims as provisional, involving readers as participants in their ratification, while showing respect for colleagues’ views (Hyland and Jiang, 2019). Hedges represent a writer’s explicit intrusion into a text to convey their personal stance (Hyland and Jiang, 2016).

Conversely, boosters are devices such as *obviously*, *clearly,* and *prove*, which allow the author to express an idea with conviction and confidence, signaling a strong statement about a state of affairs (Hyland, 1998a). Boosters function to assist authors in emphasizing certainty and suppressing alternative voices while constructing rapport by marking involvement, solidarity, and engagement with readers (Hyland, 2005a). They are also an important strategy that enables authors to emphasize the significance, uniqueness, or originality of a claim in research writing (Hyland, 2005a; Hyland and Zou, 2021).

Following Hyland and Zou (2021), who draw on Hinkel (2005) and Salager-Meyer (1994), this study classifies hedges and boosters into three types each to capture fine-grained rhetorical variation. Hedges are divided into downtoners, rounders, and plausibility hedges.

- Downtoners are typically adjectives or adverbial phrases that reduce the intensity of a claim (e.g., quite, probably, on the whole).
- Rounders express numerical approximation or imprecision (e.g., about, around, approximately).
- Plausibility hedges mainly include modals and lexical verbs that suggest a statement is based on plausible reasoning rather than evidence (e.g., could, might, and indicate).

Conversely, boosters are classified into intensity boosters, extremity boosters, and certainty boosters.

- Intensity boosters amplify the writer’s emotional strength of a statement (e.g., extremely difficult, particularly important).
- Extremity boosters underline the upper edge of a continuum (e.g., most, best, largest).
- Certainty boosters signal the author’s epistemic conviction (e.g., show, find, definite).

This categorization is adopted because it highlights subtle differences in epistemic positioning across time and disciplines. It provides a more detailed analytical framework than broader two-category models and directly aligns with the study’s research questions. This approach has also been applied by Xie et al. (2024), further supporting its validity and usefulness for examining diachronic patterns in academic discourse. Overall, epistemic positioning is crucial to the rhetorical and interactive character of research writing (Hyland, 1998a). It reflects a writer’s investment in their statements, either by conveying confidence in their factual reliability or by withholding full commitment to indicate that a claim is based on reasoning rather than established facts (Hyland and Jiang, 2016).

### 2.2 Epistemic positioning in research writing

Increasing research into epistemic positioning has been conducted in different languages and genres, most commonly in an academic register. Research on epistemic positioning has predominantly focused on comparing texts across different languages (typically English and another language; Hu and Cao, 2011; Mu et al., 2015; Mur-Dueñas, 2011, 2021) and examining differences between writers at varying proficiency levels (commonly student and expert writers; Qiu and Ma, 2019; Wang and Jiang, 2018). Studies in the first category have shown that successful academic writing in English tends to incorporate more hedges than texts in other languages, reflecting the influence of distinct linguistic and cultural norms. For example, Mur-Dueñas (2021) compared the use of hedges in English and Spanish research articles on business management and found that English texts featured a significantly higher frequency of hedges. In contrast, studies in the second category have observed that student writers often incorporate more epistemic positioning features, reflecting their tentativeness in making claims and a tendency to overgeneralize. This tendency may stem from a limited understanding of the pragmatic implications of their language choices. However, Abdollahzadeh (2019) analyzed the use of hedges in discussion sections in applied linguistics written in English by Iranian and English graduate students and professional writers, finding that student writers generally employed fewer hedges. Similarly, Dontcheva-Navratilova (2024) observed that master’s theses by Czech students contained fewer hedging expressions but slightly more boosting language than L1 expert writers. These variations in findings could be attributed to differences in the sections or types of texts analyzed and may also be closely linked to the writers’ linguistic backgrounds.

In addition, cross-disciplinary epistemic positioning studies from a synchronic view have been particularly productive and have demonstrated variations in the ways writers employ epistemic positioning not only in research articles (Lafuente Millán, 2008) but also in undergraduate essays (Li and Wharton, 2012), textbooks (Hyland, 1999), book reviews (Tse and Hyland, 2009), academic presentations (Hyland and Zou, 2021; Qiu and Jiang, 2021), and online live talks (Yuan et al., 2024).

For example, Hyland (2005b) analyzed stance markers in research articles from eight disciplines and found that hedges and boosters were more prevalent in the soft fields than in the hard fields, reflecting the underlying epistemological divergence between soft and hard fields. Similarly, Peacock (2006) investigated the use of boosters in research articles across six disciplines and found the highest frequency in linguistics, with the lowest frequency in environmental science. This finding reveals a divergent type and a narrower range of boosters in the two sciences compared to the other four soft disciplines. Moreover, Hu and Cao (2015) conducted a cross-paradigmatic and cross-disciplinary analysis of hedges and boosters in the post-method sections of research articles from applied linguistics, education, and psychology, three social science disciplines. They revealed significant differences in the use of hedges and boosters across both research paradigms and disciplines, suggesting that epistemic positioning is shaped by both methodological and disciplinary conventions. These studies show a marked variation in academic persuasion and have identified the rhetorical and social distinctiveness of disciplines (Hyland and Jiang, 2018). Although previous research has extensively examined disciplinary variation in the use of epistemic positioning, much less attention has been paid to how these features shift over time. Therefore, a longitudinal and cross-disciplinary study is essential to gain a deeper understanding of the dynamic patterns of epistemic positioning in academic writing.

Finally, research into epistemic positioning use from a diachronic perspective is a recent endeavor, yet it has yielded some noteworthy findings. Existing studies mainly focus on the changing patterns of metadiscourse resources across different disciplines (Deng et al., 2021; Hyland and Jiang, 2016, 2018) or within a single field (Gillaerts and Van de Velde, 2010; Poole et al., 2019; Xie et al., 2024). Cross-disciplinary studies commonly investigated both interactional and interactive metadiscourse resources, typically comparing patterns between soft and hard science fields. For example, Hyland and Jiang (2016, 2018) showed a corpus of research articles from applied linguistics, sociology, biology, and electrical engineering published between 1965 and 2015. This study identified a uniform decline in both hedges and boosters across soft disciplines but a general rise in hard disciplines, except for a slight decrease in boosters in biology. Similarly, Deng et al. (2021) explored the changing patterns of interactive metadiscourse and interactional metadiscourse in doctoral dissertation writing across humanities and social sciences and sciences and engineering at three time intervals (1966, 1986, and 2016), finding a substantial reduction in hedges and boosters in humanities, but a general rise in hedges and no significant decline in boosters within science disciplines. Single-discipline studies, meanwhile, offer more focused insights into epistemic positioning. Gillaerts and Van de Velde (2010) observed a consistent decline in boosters in abstract sections of applied linguistics research articles, whereas hedges increased in recent years. Xie et al. (2024) investigated the use of hedges and boosters in the discussion sections of Chinese MA theses and published research articles in applied linguistics over the past 30 years. Their findings revealed an overall downward trend over the past 30 years among both novice and expert writers, despite some fluctuations in the data. In contrast, Poole et al. (2019) examined biochemical research articles from 1972 to 2017 and found a decline in the use of hedges but an increase in the use of boosters. These divergent results may be attributed to two key factors: variations in the selected sections of the texts and disciplinary conventions under investigation. For example, abstract sections not only provide a summary of the accompanying article but also serve as an advertisement to promote it and are more likely to use boosters to enhance persuasive force (as shown in Gillaerts and Van de Velde, 2010). In contrast, the research article discussion sections primarily serve to interpret research findings and acknowledge uncertainty, often containing more hedging language, as observed by Xie et al. (2024). Disciplinary conventions also play a crucial role in shaping epistemic positioning over time. In soft disciplines such as applied linguistics, the observed overall decline of both hedges and boosters may indicate that authors move toward a more cautious and neutral expression over time. By contrast, previous diachronic studies examining various hard science fields have reported divergent patterns, with some disciplines showing increasing or decreasing use of boosters or hedges. These inconsistencies suggest that even within the hard sciences, disciplinary norms and evolving research practices influence diachronic changes in epistemic stance. Therefore, disciplinary context not only influences the stance writers prefer at a given period but also shapes how that stance shifts across decades.

Despite these contributions, existing diachronic studies on epistemic positioning still have clear limitations. First, most comparative studies include soft disciplines only through applied linguistics, while the hard disciplines selected vary widely, making the patterns less generalizable. Second, only Poole et al. (2019) and Xie et al. (2024) conducted fine-grained analysis of hedges and boosters, but both were confined to a single discipline, limiting the scope of their findings. To address these gaps, the present study undertakes a detailed diachronic analysis of epistemic positioning features across four representative disciplines, namely the soft-applied field of education, the soft-pure field of history, the hard-applied field of mechanical engineering, and the hard-pure field of physics. It examines research articles published in 1960, 1990, and 2020 to trace the evolution of hedges and boosters within and across disciplines.

## 3 Corpus and methods

We created three corpora, each consisting of 240 research articles from each of five journals, spanning four disciplines, at three distinct periods over the past 60 years: 1960, 1990, and 2020. According to Becher and Trowler (2001), we selected education, history, mechanical engineering, and physics as representatives of the soft applied, soft pure, hard applied, and hard pure domains, respectively. Four research articles were randomly selected from each of five journals for every discipline and period. These journals achieved top rankings in their respective fields based on the 2019 5-year impact factor, as reported in the Journal Citation Reports (Clarivate Analytics, formerly Thomson Reuters). The chosen journals are listed in Appendix A. Additionally, single and co-authored articles were equal except in history, where single authorship predominates. Only the main text of each selected article was retained, with abstract, tables, figures, complex equations, block quotations, references, and footnotes excluded. Each article was assigned a label in the format of “Corpus number - Discipline - Article number - Abbreviation of Journal - Article number.” For instance, “01E04-JTE02” refers to the 4th article in the 1960 corpus in education and the second article in the Journal of Teacher Education. However, for ease of reading, examples in the following text are referred to only by discipline and year. The corpus comprises a total of 240 journal articles, spanning approximately 1.3 million words, as depicted in Table 1. Our data indicate a marked increase in article length across all fields over the 60-year period, which is consistent with previous observations of growing article length in academic writing (Chen and Hu, 2020; Hyland and Jiang, 2016, 2017, 2018).

**Table 1:** Corpus characteristics.

Discipline	1960	1990	2020	Overall
Education	53,405	83,785	161,691	298,881
History	132,719	195,589	185,708	514,016
Mechanical Engineering	78,476	76,589	124,568	279,633
Physics	63,757	72,498	74,384	210,639
Total	328,357	428,461	546,351	1,303,169

**Figure 1:**
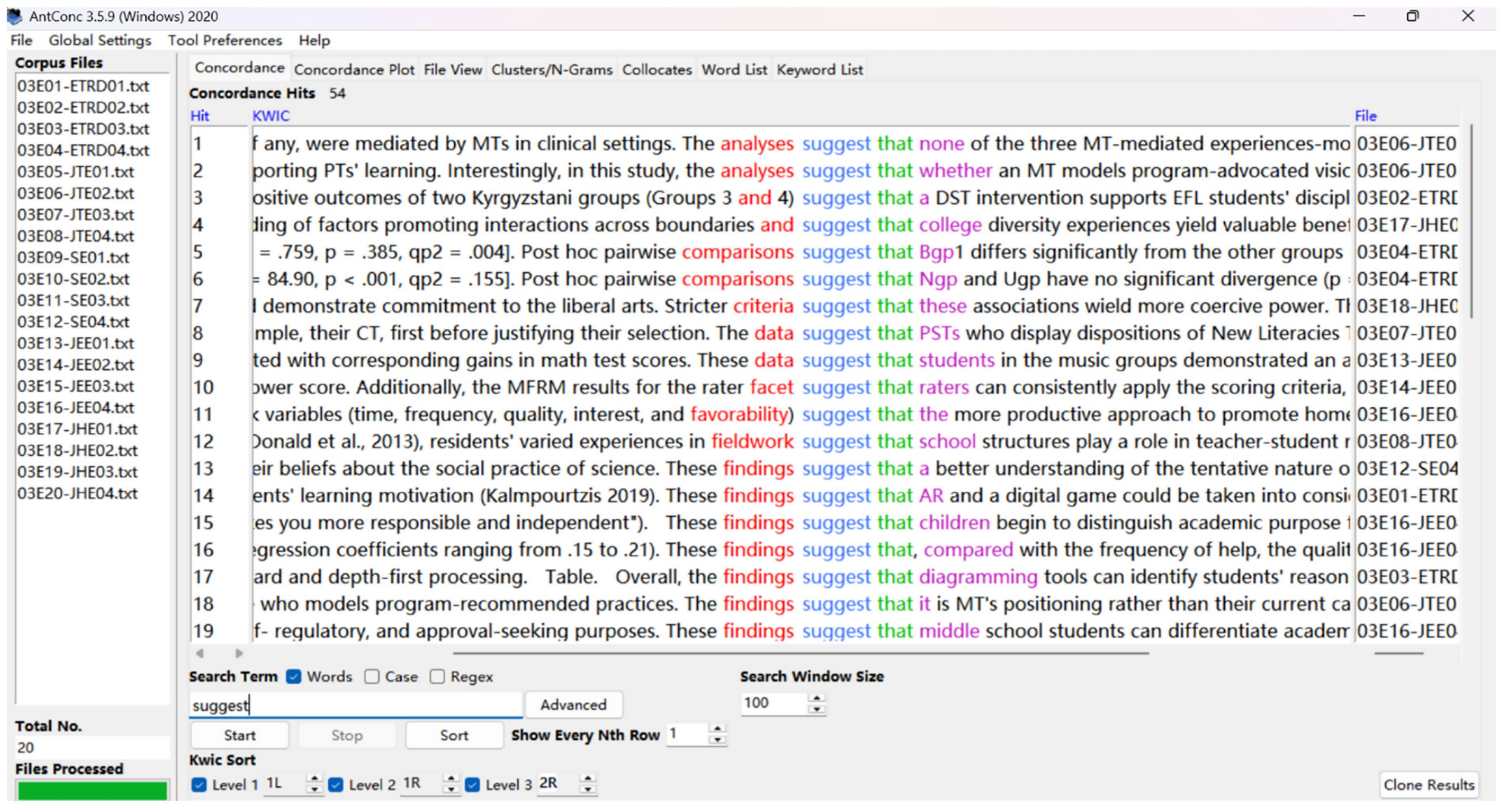
Sample of concordance lines from education in 2020.

Drawing on Hyland (2005a) framework and following the taxonomies of hedges and boosters proposed by Hyland and Zou (2021), we created a draft list of over 200 epistemic features (Appendix B) for investigation by reviewing relevant literature on hedges and boosters (Gillaerts and Van de Velde, 2010; Hyland, 2005a, 2005b; Mur-Dueñas, 2011) for reference. Then, we used the concordance software (Anthony, 2020) to search the items in the self-compiled corpora, as shown in Figure 1. Afterwards, we manually checked each retrieved concordance line to ensure that these items function as epistemic positioning in their contexts, excluding those extraneous examples from the frequencies of hedges or boosters.

For instance, in example (1), the verb *show* means *display* in a literal sense, while in example (2), *show* conveys the author’s epistemic certainty and full commitment to their findings. Thus, only (2) was coded as a certainty booster. Likewise, in example (3), *could* indicates a lack of ability or capacity to perform the prediction or report results, whereas in (4) it expresses tentative possibility or probabilistic reasoning, signaling the writers’ withdrawal of full commitment. In this case, *could* in (3) was excluded and (4) counted as a plausibility hedge. Similarly, in (5), the verb “*suggest*” signals that the conclusion is inferred from evidence and thus functions as a plausibility hedge, whereas in (6), it simply means “*put forward*” and is not an epistemic positioning device. Therefore, “*suggest*” in example (6) was excluded from the frequency calculation.

(1) They were asked to write equations, *show* all work, and complete all math problems to the best of their ability. (Edu, 1990)
(2) The results also *show* that as the channel height increases, the pressure drop decreases sharply. (Mech Eng, 1990)
(3) However, DPM *could* not predict/report nanoparticle clustering. (Mech Eng, 2020)
(4) The research outcomes *could* also be a result of the amount of time spent on video production. (Edu, 2020)
(5) Our results *suggest* that students are more situationally engaged when they are doing certain scientific practices. (Edu, 2020)
(6) Thus, residents *suggest* that NETR’s version of student teaching does not allow them much space to form relationships with students. (Edu, 2020)

To ensure the results were valid and reliable, both authors independently coded the data. Inter-coder agreement reached 95% (Kappa = 0.95, calculated via SPSS 20.0), indicating a high level of consistency between the two raters. Discrepancies were resolved through discussion until consensus was reached. For comparability across disciplines and periods, all frequencies were normalized per 10,000 words, following the standard practice in previous studies (Hyland and Jiang, 2016, 2017, 2018; Xie et al., 2024). Finally, a log-likelihood test was used to assess statistical significance, with *p* < 0.05 as the significance threshold for identifying meaningful differences.

Based on the aforementioned framework and procedures, this study examines the nuanced diachronic changes of epistemic positioning across the four disciplines over the past 60 years.

## 4 Results and discussion

### 4.1 Changes in epistemic positioning: overall results

**Figure 2:**
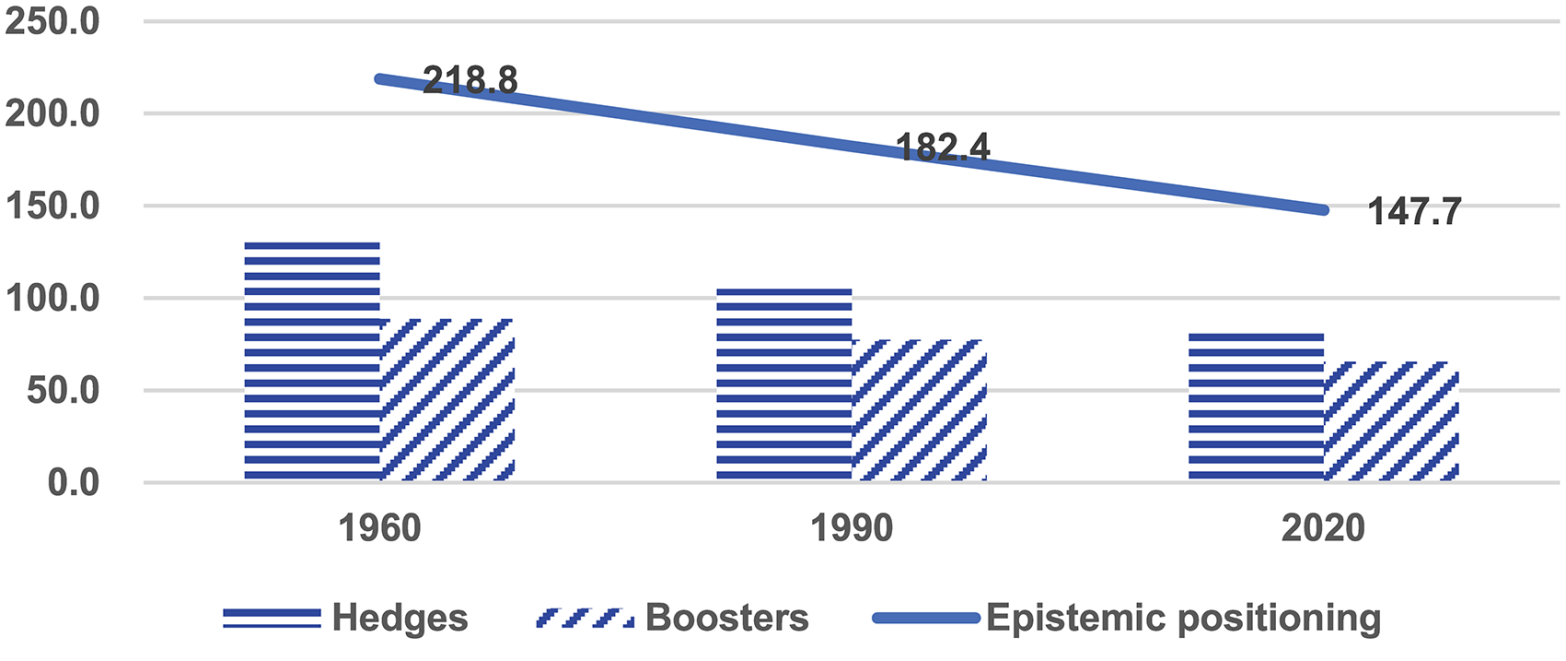
Change of epistemic positioning over time (per 10,000 words).

Overall, we found 147.7 cases of epistemic positioning per 10,000 words of text in the 2020 corpus. Figure 2 shows that epistemic positioning has dropped markedly by 32.5% (log likelihood = 579.17, *p* < 0.001) since 1960.

Thus, epistemic positioning features have declined considerably in research writing over the past 60 years. While investigating the corpus, it was found that there was a relatively substantial decrease in the use of hedges (log likelihood = 458.51, *p* < 0.001) compared to the reduction of boosters (log likelihood = 144.06, *p* < 0.001) between 1960 and 2020. Table 2 illustrates changes in the use of epistemic positioning markers in academic writing across disciplines over time, showing a decrease in the use of both hedges and boosters.

**Table 2:** Changes in epistemic positioning by disciplines (per 10,000 words).

Types	Education			History			Mech Engineering			Physics		
	1960	1990	2020	1960	1990	2020	1960	1990	2020	1960	1990	2020
Hedges	143.8	125.2	88.1	132.6	114.2	84.3	126.7	84.6	78.1	117.9	78.5	70.6
Boosters	68.7	58.6	43.9	89.7	76.2	58.6	99.4	95.4	92.9	89.9	82.9	84.0
Total	212.5	183.8	132.0	222.3	190.4	142.9	226.1	180.1	171.0	207.8	161.4	154.6

Overall, epistemic positioning features have steadily declined across all four disciplines over the 60-year period. However, the timing of major reductions differs across disciplinary domains. In the soft disciplines, particularly education and history, the decline was more pronounced between 1990 and 2020, with decreases of 28.2% (log likelihood = 95.99, *p* < 0.001) and 24.9% (log likelihood = 129.18, *p* < 0.001), respectively. In contrast, the hard disciplines, mechanical engineering and physics, experienced more significant reductions between 1960 and 1990, by 20.4% (log likelihood = 40.47, *p* < 0.001) and 22.3% (log likelihood = 39.83, *p* < 0.001), with no significant reduction between 1990 and 2020 (by 5% log likelihood = 2.22, *p* < 0.2; 4.2%, log likelihood = 1.07, *p* < 0.4, respectively). These patterns suggest a temporal shift in preferences for epistemic stances. Writers in the soft fields have exhibited a movement toward making a statement with reduced epistemic marking in the past 30 years, reflecting an increasing orientation toward a scientific approach traditionally associated with the hard disciplines (Hyland and Jiang, 2016). By contrast, the rhetorical shift in the hard sciences occurred earlier, between the 1960s and 1990s, and has since remained relatively stable. Such divergence highlights the influence of disciplinary writing norms not only on how epistemic markers are deployed, but also on when these shifts manifest over time.

On the one hand, it seems that the decline in hedges is evenly distributed across all fields over the period, by 38.8% (log likelihood = 113.34, *p* < 0.001) in education, by 36.5% (log likelihood = 170.33, *p* < 0.001) in history, by 38.3% (log likelihood = 113.99, *p* < 0.001) in mechanical engineering, and by 40.2% (log likelihood = 83.14, *p* < 0.001) in physics. However, upon closer examination of the corpus, we found that the major reductions occurred at different periods across disciplines. In the soft fields, education and history saw their most substantial declines between 1990 and 2020, by 29.7% (log likelihood = 73.09, *p* < 0.001) and 26.2% (log likelihood = 86.02, *p* < 0.001), respectively. In contrast, the hard fields experienced their sharpest drops much earlier, between 1960 and 1990, with decreases of 33.2% (log likelihood = 65.72, *p* < 0.001) in mechanical engineering and 33.5% (log likelihood = 54.39, *p* < 0.001) in physics. These were followed by minor and non-significant declines of 7.7% (log likelihood = 2.47, *p* < 0.2) and 10.1% (log likelihood = 3.08, *p* < 0.08), respectively, thereafter. This demonstrates that writers across all fields have increasingly tended to downplay their statements over the past 60 years. More specifically, the soft fields, especially over the past 30 years, have shifted toward increasing scientism, which usually dominates in the hard sciences (Glynos and Howarth, 2007; Hyland and Jiang, 2018).

On the other hand, the use of boosters has also decreased across all four fields over the past 60 years, although the decline in the two hard disciplines is not statistically significant. In the soft fields, boosters followed a pattern broadly similar to that of hedges, showing a steady decline and a pronounced drop in the past three decades. Specifically, the number of boosters decreased by 25.1% in education (log likelihood = 23.68, *p* < 0.001) and by 23.1% in history (log likelihood = 43.79, *p* < 0.001) between 1990 and 2020. In contrast, boosters in the hard sciences showed only slight and statistically non-significant decreases, with reductions of 6.6% in mechanical engineering (log likelihood = 2.13, *p* < 0.2) and 6.5% in physics (log likelihood = 1.35, *p* < 0.3). These trends suggest that soft disciplines have gradually adopted more “author-evacuated” prose, aligning with the stylistic conventions of hard-science writing (Hyland and Jiang, 2016). These disciplinary differences can be understood in light of epistemological orientations and rhetorical practice. Hard disciplines are characterized by cumulative, empirically verifiable knowledge and a higher degree of internal consensus (Becher and Trowler, 2001). Such environments encourage the use of boosters to project factual reliability and reinforce the authority of findings, with hedges serving as secondary qualifiers. This is reflected in Table 2, where boosters consistently outnumbered hedges in mechanical engineering and physics since 1990. In contrast, soft disciplines rely more heavily on interpretive reasoning and the reader’s negotiation, which historically required more explicit stance markers to involve audiences and justify claims (Hyland, 2005a). In recent decades, however, the growing internationalization of academic publishing and the influence of hard-science conventions appear to have prompted soft-discipline authors to moderate their stance and reduce overt expression of certainty, resulting in the observed decline in boosters.

Now, we know how epistemic positioning changes across disciplines over time. However, it is unclear whether the types of hedges and boosters have undergone the same changing patterns or what forms have changed significantly across disciplines. In what follows, we aim to address the questions mentioned above.

### 4.2 Changes in hedges

Hedges concern the authors’ decision to withhold full commitment to a proposition, allowing authors to negotiate with readers in a discursive space. Based on the types of hedges, we can see more explicit variations across disciplines and time. Table 3 presents plausibility hedges, which are by far the most frequent hedging items in all four fields, and rounders, which have the least frequency of hedging devices across all four disciplines and periods. It is surprising that although all three types of hedges have consistently decreased substantially across the board, their respective proportions have remained almost the same over the years, with downtoners, rounders, and plausibility hedges accounting for 34.3, 6.7, and 59% in 1960, and 33.4, 7.5, and 59.1% in 2020.

**Table 3:** Changes in hedges by disciplines (per 10,000 words).

Types	Education			History			Mech Engineering			Physics		
	1960	1990	2020	1960	1990	2020	1960	1990	2020	1960	1990	2020
Downtoners	54.1	43.4	29.1	45.1	42.0	28.7	40.1	31.7	28.3	39.4	27.7	21.1
Rounders	4.1	3.8	3.4	5.0	3.0	2.4	13.3	11.4	11.3	12.5	5.8	6.9
Plausibility	85.6	77.9	55.5	82.4	69.2	53.1	73.3	41.5	38.5	66.0	45.0	42.6
Total	143.8	125.2	88.1	132.6	114.2	84.3	126.7	84.6	78.1	117.9	78.5	70.6

Downtoners are generally adverbs, adjectives, and some phrasal forms used to reduce or soften a statement’s intensity and help writers increase their credibility while making a claim. They are used to either add precision to a new statement that is unproven (e.g., 1) or protect writers against inaccuracy (e.g., 2; Hyland and Zou, 2021).

(7) A *quite* different example may be found in colonial North America, where the original European settlers …. (His, 1990)
(8) *In most cases* the K(H) dependences were plotted at constant temperature. (Phy, 1990)

Downtoners have decreased uniformly in the four disciplines across the years. However, when looking more closely at the corpus, we find downtoners in the soft fields have shown a bigger fall, especially in the recent 30 years, with a drop of 32.9% (log likelihood = 32.04, *p* < 0.001) in education and of 31.6% (log likelihood = 47.71, *p* < 0.001) in history, while there are quite different changing patterns in the hard sciences. Downtoners in mechanical engineering have declined substantially between 1960 and 1990, by 21% (log likelihood = 7.65, *p* < 0.01), but there has been no significant reduction between 1990 and 2020, by 10.9% (log likelihood = 1.91, *p* < 0.2). However, physics has shown a nearly even distribution of drop by 29.6% (log likelihood = 13.83, *p* < 0.001) between 1960 and 1990 and by 23.9% (log likelihood = 6.61, *p* < 0.02) between 1990 and 2020. This indicates that writers in the soft fields have been increasingly using fewer downtoners to avoid the uncertainty of statements, especially between 1990 and 2020, while writers in the hard disciplines have gradually displayed a movement toward employing fewer downtoners between 1960 and 1990.

Interestingly, *possible* has remained the most frequently used downtoners across the four disciplines over these 60 years. However, their combined proportion accounted for 6.58 cases per 10,000 words in the 1960 corpus and 3.04 cases in the 2020 corpus, representing a 53.8% reduction. Moreover, the combined frequencies of the top 20 downtoners in 1960 and 2020 have also declined by 37.9% by 2020. This demonstrates that writers in all fields have moved toward expressing less uncertainty and probability in their research writing to increase the chances of publication, as publishers prefer more certain and explicit research. It could be observed that, *probably*, indicating less certainty regarding the truth of the proposition (Holmes, 1982). The top three items in both education and history in the 1960 corpus were often replaced, representing the frequency of a behavior, in both the 1990 and 2020 corpora. In addition, *probably* in both education and history, they had disappeared from the top 15 by 2020. This seems to signal that the soft disciplines have gradually shifted away from speculations of the propositions (e.g., 3) to descriptions of the information (e.g., 4) under discussion, suggesting a trend toward empirical commitments to claims.

(9) The education instructor *probably* views all his students with an eye to their potentialities for teaching. (Edu, 1960)
(10) These acts have *often* confounded legal scholars because, for much of the nineteenth and twentieth centuries …. (His, 2020)

Rounders express approximation are usually associated with quantitative data, and signal that the writer provides the figures with as much accuracy as possible (Rowland, 1995). Rounders imply the degree of precision and convey to the readers a sense that the information might be accurate, as the authors seek precision in expression and do not use exaggeratedly exact markers (Hyland, 1998b), helping make the statements more accessible and persuasive to readers (Hyland and Zou, 2021). Rounders, therefore, are generally more dominant in the measurement-based hard sciences than in the discursive soft fields, as shown in Table 3.

Rounders have also decreased in the four disciplines, but have not been evenly distributed over the past 60 years. Table 3 shows that the number of rounders has decreased by 52% per 10,000 words (log likelihood = 14.89, *p* < 0.001) in history and by 45.4% (log likelihood = 13.55, *p* < 0.001) in physics over the years. In addition, both education and mechanical engineering have declined slightly, although not significantly, with drops of 17.4% (log likelihood = 0.56, *p* < 0.5) and of 14.6% (log likelihood = 1.48, *p* < 0.3), respectively. This indicates that all fields have shown a trend toward employing fewer rounders. However, education and mechanical engineering have seen a slight decline in the use of rounders in the past 60 years. It might be assumed that writers in all disciplines seem to anticipate readers’ preference for more explicit and accurate indicators in their research writing and have gradually moved toward using fewer rounders.

The most common form in each period, except in physics in 2020, remained *about* the same; however, its total frequencies decreased from 6.03 cases per 10,000 words in 1960 and 2.01 cases in 2020, falling by 66.6% over the period, and represented 72.5% of all-rounders in 1960 and only 37.7% in 2020. The other forms of rounders, such as approximately and around, have become more common in both 1990 and 2020, with their combined frequencies per 10,000 words increasing by 41.9% between 1960 and 1990 and 23.9% between 1990 and 2020. On the other hand, the total frequencies of rounders have fallen by 35.5% (log likelihood = 27.19, *p* < 0.001) per 10,000 words over these 60 years. This suggests that writers not only declined to use round numbers but also shifted away from the forms, expressing ideas with a lack of precision through a much wider array of devices, such as “*approximately*” and “*around*,” as the period elapsed.

Surprisingly, the meaning of “*around*,” with some approach to exactness and generally used in casual conversation, as the function of rounders, never appeared in physics in 1960. However, it ranked second in 1990 and at the top in 2020. Nevertheless, “*about*,” indicating reasonably close to exactness, a more common form, the top one in rounders in physics in both 1960 and 1990, has dropped to the last place in 2020. It might be reasonable to assume that physics has undergone the biggest shift in the use of rounders across the four fields, especially in the last 30 years, since the top two rounders in the other three disciplines have remained the same in the past 60 years. On the other hand, *approximately*, suggesting a more careful calculation and a more formal item, the other three disciplines have displayed a steady increase, especially in the soft fields, with a rise of 185.9% in history and by 32.1% in education (normed to per 10,000 words) by 2020. In addition, “*about*,” the most frequently used form, has declined dramatically across all fields over these 60 years. This suggests that authors in all disciplines are inclined to express their ideas as accurately as possible. This trend aligns with a broader trend toward greater precision within a high-tech context.

(11) The second remarkable change occurs at *about* – 40 °C. (Phy, 1960)
(12) The 12QMSDW stabilized *around* (n_e_, U) = (1.75, 5) are regarded as the superposition of the 2-4QMSDW and the remaining 4QMSDW. (Phy, 2020)
(13) Each sheep yielded *approximately* four kilograms of meat and half a kilogram of wool per year, scarcely enough. (His, 2020)

Plausibility hedges indicate that a claim is based on some doubt rather than complete certainty (Rowland, 1995). They function to soften the intensity of assertions and engage readers to participate in the conversation. Plausibility also decreased uniformly across all four disciplines over the past 60 years, though the timing and extent of the decline varied between soft and hard fields. In the soft disciplines, the most significant drop occurred between 1990 and 2020, with a drop of 28.7% (log likelihood = 42.45, *p* < 0.001) in education and 23.2% (log likelihood = 40.32, *p* < 0.001) per 10,000 words in history. In contrast, the hard sciences experienced sharper declines earlier, between 1960 and 1990, with mechanical engineering declining by 43.3% (log likelihood = 68.9, *p* < 0.001) and physics by 31.9% (log likelihood = 27.4, *p* < 0.001) per 10,000 words. These findings reveal a discipline-specific temporal trend: authors in the soft sciences have only recently begun to adopt a more assertive or objectivist rhetorical stance, while writers in the hard sciences underwent this shift several decades earlier. This divergence indicates that disciplinary writing conventions not only influence the degree of epistemic caution but also the historical paths along which these conventions evolve.

The most frequently used plausibility hedges in both education and history remained *may*, *would*, *could,* and *might* by 2020. They represented 63% of all plausibility hedges in education and 77.4% in history in 1960, and 61.6 and 70.3%, respectively, in 2020. However, their combined frequencies have declined by 36.6% per 10,000 words in education and by 41.4% in history over the period. On the one hand, *may* and *would* remain the top 2 forms for both hard sciences throughout the period, but their total frequencies have sharply declined, dropping by 56.7% in mechanical engineering and 57% per 10,000 words in physics. *Suggest*, *assume*, and *indicate* were the only forms across the four fields to show a slight increasing trend among the most plausible hedges. This trend of decline in modal verbs and increase in lexical verbs across is totally consistent with previous studies (Hyland and Jiang, 2016; Poole et al., 2019; Xie et al., 2024). This suggests that writers across the disciplines not only experience a decline in the use of plausibility hedges but also a shift away from some forms. On the other hand, this indicates authors prefer to use plausibility hedges to make more speculative interpretations (e.g., 8), utilizing the uncertainty of human assessment rather than of the reliability of rational deduction or the vagaries of observed data (e.g., 9; Hyland and Jiang, 2018).

(14) More comprehensive programs, perhaps begun at an earlier age, *may* be necessary to sustain significant long-term attitude changes of this type. (Edu, 1990)
(15) The results in Fig. 24 *suggest* an increase in the extent of R_uu_ with Reynolds number, particularly close to the wall. (Mech Eng, 2020)

### 4.3 Changes in boosters

Contrary to hedges, boosters express the writers’ certainty in what they say, signaling that the writers close down possible alternatives. Based on the types of boosters, we aimed to investigate how the use of boosters has changed across the disciplines over the past 60 years.

As shown in Table 4, the use of boosters has declined significantly in the soft disciplines, and there has been no significant drop in the hard fields. However, the types of boosters exhibit a highly divergent pattern across the fields and over the years. Certainty boosters have shown a declining trend across the board, although not significantly in physics, while they still dominate all disciplines across all periods. Intensity boosters showed a notable drop in education (−27.9%, log likelihood = 4.99, *p* < 0.05), but displayed an uneven upward trend in the other three fields. Extremity boosters decreased by 37.3% in education and 22.2% in history but showed a slight increase in mechanical engineering and physics, although the increase was not significant. These patterns indicate discipline-specific preferences and diachronic variations in how writers amplify epistemic stance in academic writing.

**Table 4:** Changes in boosters by disciplines (per 10,000 words).

Types	Education			History			Mech Engineering			Physics		
	1960	1990	2020	1960	1990	2020	1960	1990	2020	1960	1990	2020
Intensity	13.3	12.9	9.6	9.8	10.9	11.9	6.8	16.2	9.7	6.4	5.2	6.5
extremity	12.9	12.8	8.1	12.7	16.6	9.9	9.3	11.2	12.2	7.1	9.4	9.4
certainty	42.5	32.9	26.2	67.2	48.7	36.9	83.3	68.0	71.0	76.4	68.3	68.2
Total	68.7	58.6	43.9	89.7	76.2	58.6	99.4	95.4	92.9	89.9	82.9	84.0

Intensity boosters enable authors to intensify their emotional strength while making a statement. They do not involve epistemic commitment but add affective color to the statements, functioning roughly like attitude markers, although doing so by raising the voice rather than conveying an attitude (Hyland and Zou, 2021). As shown in Examples 10 and 11, writers seek to convey their strong stances by using *extremely* and *highly*, thereby expressing a high degree of certainty, involving the readers in their statements, and making them accept what is said as a given (Athanasiadou, 2007).

(16) A consoling faith that the Lord would provide for all those he sent was *extremely* common among pre-limiters. (His, 1990)
(17) The comparisons show that the present approach produces *highly* accurate results of displacement components at the critical locations in the beam. (Mech Eng, 2020)

Unexpectedly, intensity boosters fell by 27.9% (log likelihood = 4.99, *p* < 0.05) per 10,000 words only in education while rising 43.8% (log likelihood = −5.08, *p* < 0.05) in mechanical engineering, with a slight growth of 21.4% (log likelihood = −3.15, *p* < 0.08) in history and almost no change (+0.3%) in physics over the past 60 years. This pattern suggests that discipline-specific rhetorical adjustments occur over time. Education, as a soft-applied discipline rooted in interpretive inquiry and policy discussion, has increasingly shifted toward neutral, depersonalized prose, likely influenced by the global dominance of hard-science publishing practices (Hyland and Jiang, 2016). Mechanical engineering, by contrast, is a hard-applied field, where research outcomes often compete for industrial recognition and funding. In such a competitive, application-oriented environment, amplifying claims through intensity boosters highlights novelty, technical superiority, and practical relevance, explaining the marked upward trend. History, as a typical soft and pure discipline, exhibits only a modest growth in intensity boosters, perhaps reflecting an attempt to increase argumentative weight and highlight the significance of interpretive contributions in a globalized scholarly market. Physics, as a paradigmatic hard and pure discipline, continues to adhere to rigid empirical reporting norms, leaving little room for fluctuation in intensity boosters and thus showing rhetorical stability over time (Becher and Trowler, 2001). Overall, these discipline-specific trajectories suggest that diachronic shifts in stance-taking are uneven and arise from the interplay of disciplinary knowledge practices, evolving publication norms, and broader socio-academic pressures, rather than from a uniform trend across all fields.

*Significantly*, it remained the most frequently used intensity booster in education throughout, although its frequency per 10,000 words has dropped the most of all intensity boosters by 66.1% by 2020, accounting for 7.1 cases in 1960 but 2.4 cases per 10,000 words in 2020. *Especially* and *particularly* remain the top two choices in history, and their combined frequencies have increased by 64.7% per 10,000 words, occupying 4.4 cases in 1960 and 7.3 cases per 10,000 words in 2020. It might be assumed that the most preferred forms of intensity boosters in the soft fields have not changed significantly over the past 60 years, and their differences in preferred use of intensity boosters represent variations in discipline culture. On the other hand, *significantly* refers to something in a sufficiently great way as to be worthy of attention (e.g., 12), while *especially* and *particularly* are used to emphasize something to a higher degree than usual or average (e.g., 13 and, e.g., 14). All these three forms seek to impress, influence, and persuade readers to accept a claim.

(18) Although Tables IV to VII do not show the results, group 5 scored *significantly* higher than any group below it, group 4 scored *significantly* higher than any group below it, …. (Edu, 1960)
(19) This, of course, made the enforcement of discipline *especially* hard. (His, 1990)
(20) Victims of atrocities who seek asylum are *particularly* vulnerable to having their information used against them. (His, 2020)

Contrary to the soft knowledge fields, the most common intensity boosters in both of the hard sciences have shifted greatly. *Particularly*, ranking first in mechanical engineering in 1960, its share decreased by 29.6% per 10,000 words by 2020, dropping from 32% of all intensity boosters in 1960 to 15.7% in 2020. However, both “*especially*” and “*significantly*” increased by 194% by 2020 and have become engineers’ most preferred choices. Similarly, *especially*, the top 1 intensity booster in physics in 1960 declined by 71.4% by 2020, from 22% of all intensity boosters in 1960 to only 6.3% in 2020. While *significantly* increased by 285.7% by 2020, it has become the most popular among physicists, accounting for 9.8% of all intensity boosters in 1960 and 37.5% in 2020. This suggests that the hard sciences have not only shown a trend toward using more intensity boosters but also shifted toward the most commonly used forms to overtly engage and persuade the readers.

(21) Differences in chip formation associated with different fluids become *particularly* evident when the specimen is polished metallographically before cutting rather than after. (Mech Eng, 1960)
(22) For slow electrons, δk^el^_y_ is *significantly* larger than the Kapitza-Dirac diffraction orders of 2 k_0_ and can therefore be easily retrieved. (Phy, 2020)

Extremity boosters are used to identify the high end of a continuum and assist authors in involving readers and removing any doubt about the statements (Hyland and Zou, 2021). They are an important strategy for impressing and influencing readers’ understanding in academic writing. They function to heighten the force of statements, as shown in the following examples:

(23) Thus, it makes sense that one of *the greatest* insults for a man of honor was to have his nose pulled or tweaked. (His, 1990)
(24) Raman spectra also support this finding with *the highest* TiB_2_ and CrN peak intensities measured for coating-C. (Mech Eng, 2020)

Extremity boosters have shown a declining trend in both of the soft fields but a modest growth, albeit not significant, in the hard sciences. Extremity boosters have fallen by 37.3% (log likelihood = 9.31, *p* < 0.01) in education and by 22.2% (log likelihood = 5.46, *p* < 0.05) in history. However, they have risen slightly by 31.2% (log likelihood = 3.74, *p* < 0.06) in mechanical engineering and by 33.3% (log likelihood = −2.31, *p* < 0.2) in physics over the past 60 years. This suggests that the soft knowledge fields have shown a trend toward avoiding intrusion into the text to seek an objective and scientific approach, while the hard sciences move in the opposite direction, toward involving and persuading readers overtly.

*Most* and *best* were the most preferred extremity boosters in education and history across all the periods, although their combined frequencies have reduced by 32% in education and by 17.5% per 10,000 words in history. Both these items are used to express an extreme or high degree of quality. They enable authors to convey a strong stance on a topic under discussion and impress and facilitate readers’ understanding. Additionally, we found that historians use a much wider array of forms than writers in the other disciplines. Those forms, such as *earliest*, *latest*, *oldest,* and *youngest,* are highly discipline-specific and consistently far more common in history, whereas they are rarely found in the other three fields.

(25) Quality of delivery has been shown to impact the effect of EBPs on desired outcomes and is arguably *the most* important, yet also *the most* difficult aspect of fidelity to achieve. (Edu, 2020)
(26) Among the extant narratives on the 1,683 raid, the testimony of Fray Juan de Avila offers *the best* example of the need to reconsider the positionality of Veracruz’s residents. (His, 2020)
(27) Thus, the evidence of the chief topic of the Gest, which is *the earliest* surviving version of the legend …. (His, 1960)

*Most* was also the most frequently used in both mechanical engineering and physics, although it has declined slightly in physics (−14.3%) and remained almost unchanged in mechanical engineering (−1% per 10,000 words). *Nearest* and *highest* never appeared in physics in 1960 and occurred only on a few occasions in 1990; however, both these items have ranked in the second and third place, respectively, by 2020. This suggests that the most common forms in physics have undergone the greatest shift across the disciplines over the past 60 years. On the other hand, the *lowest* and *highest* in mechanical engineering have increased significantly by 482.7 and 530% (per 10,000 words), respectively, by 2020. *Nearest*, *highest,* and *lowest* are far more common in the hard disciplines than in those of the soft knowledge fields. These forms usually collocate with some numerical materials in the hard disciplines. The notable increase in these forms within the hard sciences suggests that scientists are increasingly marking extremity boosters related to numerical data. This practice reinforces their confidence in their judgments and helps to preclude alternative interpretations effectively (Hyland, 2012).

(28) *The highest* recorded T_c_ of element superconductors is 29 K in calcium (Ca), which was found at a pressure exceeding 200 GPa. (Phy, 2020)
(29) The L05 plot shows that they reached *the lowest* level, with a maximum of approximately 2.5°, indicating that the workpiece became the most rounded at this stage. (Mech Eng, 2020)

Finally, certainty boosters allow authors to emphasize their epistemic conviction in statements (Koutsantoni, 2004). By conveying a clear and strong stance toward the certainty or truth of a proposition, writers can demonstrate involvement and solidarity with their readers, stress shared knowledge within scientific communities, and engage directly with their readers (Hyland, 2004). Table 4 depicts that certainty boosters dominate the frequencies in both the soft and hard disciplines throughout the period, although their overall frequency has shown a declining trend over time. Notably, certainty boosters are more prominent in the hard sciences, likely because these fields rely on data and experiments, leading writers to express greater confidence in their findings or results.

Certainty boosters have fallen uniformly in all four disciplines over the past 60 years, although the decrease in physics is not significant. More specifically, certainty boosters have declined by 38.4% per 10,000 words (log likelihood = 32.6, *p* < 0.001) in education, by 45.1% (log likelihood = 141.08, *p* < 0.001) in history, by 14.8% (log likelihood = 9.62, *p* < 0.01) in mechanical engineering, and by 10.8% (log likelihood = 3.22, *p* < 0.08) in physics. This means writers have displayed a preference for fewer marking certainty boosters across all the disciplines and time, especially in the soft knowledge fields.

*Must*, the primary model of inferential certainty, has reduced uniformly in the four fields, except for a minor increase in education. However, the overall proportion of lexical verbs (e.g., *found*, *shown*, *demonstrate,* and *prove*) among all certainty boosters has consistently increased across all four disciplines in our corpus, representing 38.3% per 10,000 words of all certainty boosters in education, 16.7% in history, 59.7% in mechanical engineering and 51.3% in physics in 1960 and 61.6, 20, 78.1, and 59.6%, respectively, in 2020. This change indicates an important shift that authors in all fields seek to express their claims, from inferential certainty to more objective, data-supported assurances (Hyland and Jiang, 2016, 2018). This finding is entirely consistent with the previous studies, such as Hyland and Jiang (2018), Poole et al. (2019), and Xie et al. (2024).

(30) Experiments suggested that there *must* be a tremendous amount of cross-connecting of heat exchangers in the body. (Mech Eng, 1960)
(31) Research in teacher observation has *shown* that teacher performance can vary depending on the time of year and based on the students in class. (Edu, 2020)

On the other hand, *the fact that* and *in fact* were used to reinforce an assertion and ranked in the top five frequently used certainty boosters in 1960 in education. However, these two forms had disappeared from the top 20 and were replaced by lexical verbs, such as “*shown*” and *demonstrated*, by 2020. *Indeed*, used to emphasize a fact, it remained the most popular device in history throughout, while its frequency had reduced by 28.5% by 2020. In addition, *indeed* is not commonly used in the other three disciplines, representing a more discipline-specific item. Finally, the forms *shown*, *found*, *shows*, and *show* were the most preferred forms by the engineers and physicists throughout. This appears to indicate that authors in both the soft and hard disciplines have shifted toward making fewer explicit assertions but more objective and data-supported commitments. This style has traditionally dominated the hard sciences. These choices are consistent with a more significant trend toward increasing scientism in a more competitive publication marketplace.

(32) This somewhat surprising finding may be due to *the fact that* the total distribution for subjects on the Taylor Anxiety Scale clustered rather heavily about the median. (Edu, 1960)
(33) *Indeed*, if anything, as we have seen, such grievances are apt to be associated with the absence of risings altogether. (His, 1990)
(34) We *found* that the 2QH consists of the two types of vortices and antivortices. (Phy, 2020)

## 5 Conclusion

In this study, we have explored changes in the use of epistemic positioning in four representative disciplines over the past 60 years. Drawing on Hyland and Zou (2021) categories of hedges and boosters, and based on a 1.3 million-word corpus of research articles across four disciplines over 60 years, we addressed three core questions.

First, with respect to the forms and frequency of epistemic positioning, we observed an overall decline in the use of epistemic positioning markers, including hedges and boosters, across all four disciplines over the past 60 years. This trend is especially pronounced in soft knowledge fields over the last three decades, while in the hard sciences, the decrease occurred mainly between 1960 and 1990. Hedges and their types have displayed a similar trend to the changes in epistemic positioning. Boosters have also steadily decreased across all four fields, although the decline in the hard sciences is not significant. However, the types of boosters have displayed a divergent changing pattern, especially in the hard fields. All types of boosters have significantly decreased in the soft knowledge fields, except for intensity boosters, which have shown a slight increase over time. In the hard sciences, while certainty boosters have declined, both intensity boosters and extremity boosters have increased over time. Our data suggest that writers have gradually shifted from modal verbs (e.g., *may*, *must*) to lexical verbs (e.g., *suggest*, *show*, *demonstrate*) over the period. This pattern aligns with Poole et al. (2019), Xie et al. (2024), and Hyland and Jiang (2016), who all reported a shift from modal to lexical stance markers in their respective corpora, reflecting a broader move toward explicit, evidence-based persuasion in academic writing.

Second, regarding the rhetorical functions of epistemic positioning, the results indicate that hedges and boosters serve as critical tools for projecting confidence or caution, aligning with disciplinary expectations, and persuading readers. The observed reduction in epistemic markers, particularly in the soft fields, may reflect an ongoing tendency toward objectivity and “scientization” in academic writing (Hyland and Jiang, 2021). This aligns with Biber and Gray (2016) observation that stance features in scientific academic writing increasingly favor implicit stance over explicit grammatical marking. Meanwhile, the modest increase of extremity boosters and intensity boosters in the hard sciences appears to signal authors’ efforts to impress and engage readers more overtly.

Finally, in addressing the variation of epistemic positioning across disciplines and time, our data point to a gradual convergence in rhetorical practices between soft and hard disciplines. In soft disciplines, both hedges and boosters have steadily declined, indicating a shift toward more assertive and empirically oriented expression. In the hard sciences, writers maintain an overall cautious stance but increasingly rely on lexicalized strategies, with intensity and extremity boosters showing a rising trend. These patterns partly support Hyland and Jiang (2016), who observed a marked decline of hedges and boosters in soft disciplines and a general rise in hard sciences except for biology. Our hard-science results, however, show a slight overall decline in boosters, likely due to the inclusion of physics, a paradigmatic hard-pure field where reporting is highly standardized and leaves limited room for overt emphasis. Moreover, our findings partly align with Poole et al. (2019), who found a diachronic decline in hedges but an overall increase in boosters in biochemistry. The divergence from their study likely reflects differences in disciplinary focus and corpus scope: they examined biochemistry alone, where competition for novelty and impact may encourage persistent booster use, whereas our inclusion of both a hard-applied field (mechanical engineering) and a hard-pure field (physics) reveals more nuanced diachronic trajectories. Similarly, our soft-discipline results are consistent with Xie et al. (2024), who reported a sustained decline of both hedges and boosters in applied linguistics discussion sections. Our full-text analysis of education and history shows a similar downward trajectory for hedges over the decades. Together, these results suggest that soft disciplines have experienced a general decline in epistemic marking and that across all fields, academic writing has gradually moved toward a higher level of scientization (Degaetano-Ortlieb and Teich, 2019; Seoane and Hundt, 2017), with hedges declining and epistemic stance becoming increasingly explicit, evidence-driven, and lexically realized. The disciplinary boundary between the soft and hard disciplines appears to have become progressively more blurred over time.

We acknowledge the limitations of our study. On the one hand, only four disciplines were selected as the representatives of changing patterns of disciplinary research writing. Therefore, we should exercise caution when generalizing the results to all disciplines. In the future, similar studies conducted in a broader range of disciplines could help validate our findings and assess their generalizability. Conversely, expert interviews could be incorporated into future research to help substantiate our tentative claims regarding the relationship between social and discoursal changes.

We believe, however, that our study has pedagogical implications for instruction in English for academic purposes (EAP) and research publications. First, EAP instructors should raise students’ awareness of discipline-specific rhetorical norms. Based on our findings, students in soft disciplines should focus on reducing unnecessary hedging and practicing evidence-based, assertive writing, while science students should be encouraged to use boosters strategically to strengthen their claims without overstating certainty. Second, EAP curricula can incorporate corpus-informed tasks that compare epistemic stance markers across disciplines and periods. These activities can help students understand how rhetorical conventions evolve in relation to disciplinary norms and broader sociocultural shifts, while also cultivating students’ critical thinking in research writing. Finally, our study may help novice academics understand the changing patterns of epistemic positioning features in their scientific communities and highlight the importance of aligning their writing with evolving disciplinary epistemologies and social practices.

## Data Availability

The original contributions presented in the study are included in the article/Supplementary material, further inquiries can be directed to the corresponding author.
