# Supplementary material for: “We find that…” changing patterns of epistemic positioning in research writing
Source: Front Psychol. 2025 Sep 26;16:1634848. doi: 10.3389/fpsyg.2025.1634848 (PMC12513368; doi:10.3389/fpsyg.2025.1634848)
Supplement: Supplementary file 1 [file Supplementary_file_1.docx]

Appendix A. Journal list

**Education**

Journal of Teacher Education (1950-)

Science Education (1916-)

Educational Technology Research and Development (1953-)

The Journal of Experimental Education (1932-)

The Journal of Higher Education (1930-)

**History**

American Historical Review (1895-)

Hispanic American Historical Review (1918-)

Past and Present (1952-)

Historical Journal (1958-)

Journal of Modern History (1929-)

**Mechanical Engineering**

International Journal of Machine Tool Design and Research (1961-1986) continued as International Journal of Machine Tools and Manufacture (1987-)

International Journal of Heat and Mass Transfer (1960-)

International Journal of Mechanical Sciences (1960-)

Wear (1957-)

Journal of Basic Engineering (1959-1972) continued as Journal of Fluids Engineering (1973-)

**Physics**

Physical Review Letters (1958-)

Annals of Physics (1957-)

Journal of the Physical Society of Japan (1946-)

Canadian Journal of Physics (1951-)

Journal of Experimental and Theoretical Physics (1955-)

Appendix B. Epistemic positioning

**Hedges**

1. **downtoners**

almost

certain

certain amount

certain extent

certain level

comparatively

doubtful

frequently

from this perspective

generally

in a sense

in most cases

in most instances

in my view

in our opinion

in our view

largely

likely

mainly

maybe

mostly

often

on the whole

perhaps

plausibly

possible

possibly

presumable

presumably

probable

probably

quite

relatively

slightly

sometimes

somewhat

to my knowledge

to some degree

to some extent

typical

typically

uncertain

unclear

unlikely

usually

1. **rounders**

about

approximately

around

roughly

1. **plausibility hedges**

appear

appeared

appears

argue

argued

argues

assume

assumed

claim

claimed

claims

could

couldn’t

estimate

estimated

feel

feels

felt

guess

indicate

indicated

indicates

may

might

ought

postulate

postulated

postulates

seems

should

suggest

suggested

suggests

tend to

tended to

tends to

would

wouldn’t

**Boosters**

1. **intensity boosters**

entirely

especially

extremely

fully

highly

particularly

significantly

strongly

truly

1. **extremity boosters**

best

biggest

blackest

brightest

busiest

cheapest

clearest

closest

commonest

darkest

deepest

earliest

easiest

eldest

faintest

farthest

fastest

fewest

fiercest

finest

finest

fittest

fullest

furthest

gentlest

greatest

hardest

highest

hottest

largest

latest

longest

loudest

lowest

meanest

most

narrowest

nearest

newest

oddest

oldest

poorest

readiest

richest

safest

sharpest

shortest

simplest

slowest

smallest

smoothest

softest

stiffest

straightest

strictest

strongest

thinnest

toughest

weakest

wealthiest

widest

youngest

1. **certainty boosters**

actually

always

apparent

apparently

believe

believed

believes

beyond doubt

certain

certainly

clear

clearly

conclusively

decidedly

definite

definitely

demonstrate

demonstrated

demonstrates

establish

established

evident

evidently

find

finds

found

in fact

indeed

indisputable

indisputably

know

known

must (possibility)

never

no doubt

obvious

obviously

of course

prove

proved

proves

really

show

showed

shown

shows

sure

surely

the fact that

think

true

undeniable

undeniably

undoubtedly
